# The role of allogeneic stem-cell transplant in myelofibrosis in the era of JAK inhibitors: a case-based review

**DOI:** 10.1038/s41409-019-0683-1

**Published:** 2019-09-18

**Authors:** Mario Tiribelli, Francesca Palandri, Emanuela Sant’Antonio, Massimo Breccia, Massimiliano Bonifacio

**Affiliations:** 1 Division of Hematology and Bone Marrow Transplantation, Department of Medical Area, University of Udine, Udine, Italy; 2 Institute of Hematology “L. and A. Seràgnoli”, Sant’Orsola-Malpighi University Hospital, Bologna, Italy; 3 Department of Oncology, Division of Hematology, Azienda USL Toscana Nord Ovest, Lucca, Italy; 4 Division of Cellular Biotechnologies and Hematology, University Sapienza, Roma, Italy; 5 Section of Hematology, Department of Medicine, University of Verona, Verona, Italy

**Keywords:** Myeloproliferative disease, Medical research

## Abstract

Allogeneic hematopoietic stem-cell transplantation (HSCT) is, at present, the only potentially curative therapy for myelofibrosis (MF). Despite many improvements, outcomes of HSCT are still burdened by substantial morbidity and high transplant-related mortality. Allogeneic transplant is generally considered in intermediate-2 and high-risk patients aged <70 years, but the optimal selection of patients and timing of the procedure remains under debate, as does as the role of JAK inhibitors in candidates for HSCT. Starting from a real-life clinical case scenario, herein we examine some of the crucial issues of HSCT for MF in light of recent refinements on MF risk stratification, data on the use of ruxolitinib before and after transplant and findings on the impact of different conditioning regimens and donor selection.

## Introduction

Myelofibrosis (MF) is a myeloproliferative neoplasm that may present as primary (PMF) or as the evolution of a previous polycythemia vera (PV) or essential thrombocythemia (ET), in which case it is referred to as secondary myelofibrosis (SMF) [1]. MF is a clonal stem-cell process that gives rise to bone marrow fibrosis, extramedullary hematopoiesis, frequent splenomegaly and anemia, constitutional symptoms, and cachexia; its clinical course is defined by a tendency toward leukemic progression and shortened survival [2].

Patients with intermediate- or high-risk disease are candidates for therapy that may include conventional drugs (e.g., hydroxyurea), transfusions, splenectomy or, more recently, JAK inhibitors such as ruxolitinib. These treatments have the major aim of improving both symptoms and overall survival, as well as quality of life [2]. However, allogeneic hematopoietic stem-cell transplantation (HSCT) currently remains the only therapy that may modify the natural history of MF [3]. As a consequence, the number of allogeneic HSCTs performed for MF has been increasing over the past years, despite the approval of JAK inhibitors [2, 4]. Notwithstanding, HSCT is associated with high treatment-related morbidity and mortality, particularly in older adults, where the risk of the procedure may outweigh the risk of disease. Moreover, HSCT requires both good performance status and a suitable stem-cell donor [4]: in recent years, the number of patients undergoing transplants using matched unrelated donors (MUD) and employing reduced-intensity conditioning (RIC) regimens has increased significantly, representing more than two-thirds of all HSCTs [5]. Nevertheless, rates of mortality and relapse related to transplant remain high, thus posing a major challenge to hematologists. Herein, a case-based review is used as the basis to briefly review the role of HSCT in MF in the current era of JAK inhibitors.

### Case presentation

A 51-year-old woman was admitted to the emergency department in December 2003 for balance disorder and was found to have extreme thrombocytosis (platelets 2667 × 10^3^/µL) and mild leukocytosis (WBC 19.0 × 10^3^/µL with 89% neutrophils). Bone marrow biopsy and aspirate were consistent with ET, karyotype was 46XX, and molecular analysis was negative for BCR/ABL. The patient was treated with hydroxyurea with partial hematologic remission; in 2004 anagrelide therapy was started, with complete normalization of platelet counts and without significant toxicity.

Starting August 2011 progressive thrombocytopenia and splenomegaly developed, and in November 2013 a bone marrow biopsy documented progression to SMF. The patient was negative for the JAK2 V617F mutation, and the IPSS score was 1 (circulating blasts 4%), corresponding to an intermediate-1 risk group. A few months later, the decision was made to enroll the patient in a protocol testing ruxolitinib in MF (JUMP trial, NCT01493414). Complete blood count (CBC) was: Hb 10.9 g/dL, platelets 92 × 10^3^/µL, WBC 19.0 × 10^3^/µL (neutrophils 72%, blasts 3%); spleen was palpable at 7 cm below the left costal margin (LCM). Electrocardiogram revealed a previous anteroseptal myocardial infarction and first-degree atrioventricular block. Ruxolitinib was started at 5 mg BID on April 2014; at the time of initiation, the patient had no relevant symptoms (DIPSS score of 1 due to circulating blasts). After 6 months of therapy, spleen size was unchanged, and the dose of ruxolitinib was increased to 10 mg BID, without any significant reduction in spleen size at any of the subsequent evaluations. However, the ruxolitinib dose was not further escalated due to platelet counts <100 × 10^3^/µL.

In October 2016, 2.5 years after starting ruxolitinib, at which time the patient was 65 years old, the spleen was palpable at 9 cm below LCM and CBC was as follows: Hb 9.8 g/dL, platelets 89 × 10^3^/µL, WBC 34.9 × 10^3^/µL (neutrophils 48%, blasts 8%). Bone marrow biopsy revealed grade 3 fibrosis. The DIPSS score was 4 and the risk group was intermediate-2. The patient exited the formal protocol, but continued ruxolitinib therapy at 10 mg BID with the addition of hydroxyurea 1 g/day due to leukocytosis and increasing splenomegaly.

In August 2017, at the age of 66, CBC was substantially unchanged and spleen size had further increased; DIPSS score was 5 (high risk). Calreticulin (CALR) gene analysis revealed a type 2 mutation. An echocardiogram and pulmonary function tests were performed, and the patient was evaluated by the allogeneic HSCT team in October 2017.

Due to the lack of a sibling donor, a search for a MUD started in November 2017, while the patient continued treatment with ruxolitinib and hydroxyurea. The patient began transfusions with red cells in November 2017, and in February 2018 she experienced repeated episodes of congestive heart failure requiring cardiovascular therapy. In March 2018, bone marrow biopsy showed grade 3 fibrosis and osteosclerosis. The patient now required continuous transfusion with 4 units of red cells per month; spleen size was stable, but abdominal ultrasound revealed portal and splenic vein thrombosis that prompted introduction of heparin therapy.

In April 2018, an 8/8 HLA-MUD was identified. Pretransplant comorbidity index (HTC-CI) score according to Sorror [6] was 5 (congestive heart failure, reduced DLCO, depression). In June 2018, the patient underwent allogeneic HSCT with PB-derived stem cells; harvest consisted of 5.8 × 10^6^/kg CD34+ cells and 52.2 × 10^7^/kg CD3+ cells. The dose of ruxolitinib was quickly tapered starting from 7 days before and stopped the day before the start of the conditioning regimen. The non-myeloablative conditioning (MAC) regimen consisted of fludarabine 30 mg/m^2^ on day −8 to day −3 and thiotepa 6 mg/kg for 2 doses on days −4 and −3, while prophylaxis for graft-versus-host disease (GvHD) included cyclosporine A, short-course methotrexate, and ATG thymoglobulin (3.5 mg/kg for two doses). Prophylaxis for Epstein Barr virus consisted of rituximab 200 mg/m^2^ on day −2.

The posttransplant period was characterized by suspected veno-occlusive disease, with jaundice, weight gain, and abdominal discomfort, which was treated with defibrotide for 18 days. The patient did not develop acute GvHD. Neutrophil recovery (ANC > 1000) was documented on day +17, while platelet recovery (PLT > 30,000/mmc) was attained only on day +90; due to poor graft function, GCSF and transfusions were needed in the months following SCT. Posttransplant chimerism analysis performed on peripheral blood CD3+ cells at 1, 3, and 4 months after SCT showed ≥95% of donor cells.

At last follow up, 5 months after HSCT, the patient was in stable conditions, with no signs or symptoms of GvHD. The spleen was palpable at 5 cm from LCM and CBC was as follows: Hb 8.5 g/dL, platelets 53 × 10^3^/µL, WBC 3.6 × 10^3^/µL (neutrophils 60%, lymphocytes 20%, no precursor nor blasts); transfusion of red cells is needed about once weekly.

## Discussion

**Fig. 1:**
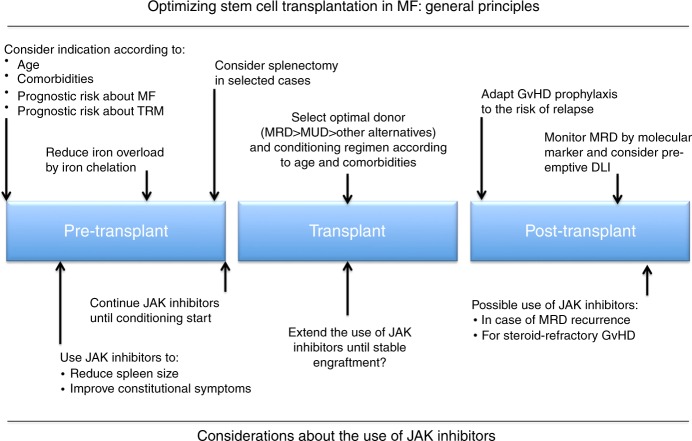
Key points in optimization of stem-cell transplantation in myelofibrosis patients. MF myelofibrosis, TRM transplant-related mortality, MRD marrow-related donor, MUD marrow-unrelated donor, GvHD graft-vs-host disease, MRD minimal residual disease, DLI donor lymphocyte infusion

The present case report allows for several observations on management of MF in the era of JAK inhibitors, as summarized in Fig. 1.

### Pretransplant evaluation: prognostic scores and considerations about use of JAK inhibitors

The mean age of patients diagnosed with MF is more than 65 years [7], and the age of the patient described herein is in line with this affirmation. At about 8 years after initial diagnosis of ET, progressive thrombocytopenia and splenomegaly were observed, and bone marrow biopsy documented progression to SMF. The management of SMF is almost identical to that of de novo PMF [8], despite the biological differences between primary and secondary MF. Initially, the patient’s DIPSS score was 1 with risk group intermediate-1 and, accordingly, HSCT was not considered to be a frontline therapeutic option, since it was largely performed in younger, fit patients with higher risk disease [9]. Moreover, while some studies have identified that subclonal nondriver mutations (i.e., ASXL1, EZH2, IDH1/2, and SRSF2) have an adverse effect on overall and leukemia-free survival [10, 11], and therefore may be used as a decisional tool for transplant (MIPPS70 [12]), this kind of analysis was not available in clinical practice in 2013, and the only information about the mutational status of our patient was the negativity for JAK2 V617F. It should also be pointed out that the prognostic impact of subclonal mutations has been mostly studied in cases of PMF, while its relevance in SMF has not yet been demonstrated [13]. Indeed, given the older age and likely coexistence of medical conditions, most patients are not considered for HSCT because of concerns regarding treatment-related toxicity [2], and some studies have reported decreased survival in patients older than 55 years of age [14, 15]. Based on these considerations, the patient was proposed enrollment in a protocol for ruxolitinib treatment, due to the presence of splenomegaly [16]. A similar approach has also been recently recommended by a panel of experts from the European LeukemiaNet and the Italian Society of Hematology [17].

After almost 3 years on ruxolitinib at what was considered to be the maximal tolerable dose, splenomegaly and thrombocytopenia were still evident and even considering that the patient was 66 years old, she was referred to evaluation by an allogeneic HSCT team, since her DIPSS score had increased to 4, corresponding to an intermediate-2 risk group. There is some discrepancy on the utility of DIPSS score in predicting outcomes [2]. While some studies have shown better outcomes for patients transplanted with a lower DIPSS score [18], patients with low-risk disease are generally not considered for transplant because survival rates appear to be higher with pharmacologic and supportive therapy, at least in the pre-ruxolitinib era [2, 19]. In addition, a clear distinction has to be made between the prognostic score related to MF itself, and the prognostic score about transplant risk. Prognostication about disease evolution can be made using simple clinical variables (IPSS or DIPSS score, [20, 21]) or considering biological characteristics such as cytogenetic (DIPSS-plus, [22]), driver mutations (MYSEC-PM, [23]) or the combination of driver and subclonal mutations (MIPSS-70, [12]). It is presently unclear which score is more accurate in defining the indication for transplant, particularly when a patient falls in categories with markedly different survival expectations. Retrospective classification of our patient revealed that at the time of SMF diagnosis she was at intermediate-2 risk according to MYSEC-PM, and at intermediate-1 risk according to DIPSS, with a projected survival of 4.5 and 14.1 years, respectively. This kind of discrepancy between risk models may occur in up to 50% of patients with SMF [24] and poses a significant challenge in transplant indication, since EBMT/ELN recommendations suggest that HSCT should be offered only to eligible patients whose survival is expected to be <5 years [13]. In such cases, we believe that information from MYSEC-PM, the only scoring system specifically developed for SMF, should outweigh the risk assessment obtained from IPSS, DIPPS, DIPSS-plus or MIPSS70, which were developed in patients with PMF and are less accurate in discriminating different prognostic groups in SMF patients [25, 26]. Moreover, we have to bear in mind that all the presently available prognostic scores have been built on cohorts of patients who were not exposed to novel drugs such as ruxolitinib, and the weight of treatment-related clinical changes on single prognostic variables compared to MF-related changes is unclear. For example, many patients on ruxolitinib develop anemia (+2 points according to the DIPSS prognostic model) but ameliorate systemic symptoms (−1 point), therefore potentially changing several times over months their prognostic group. Altogether, these considerations reflect the uncertainty about prognostic scores alone in defining transplant indication. In this regard, it should be underlined that the predictive efficacy of MYSEC-PM score in ruxolitinib-treated patients has been validated in a retrospective study [24] and that two recent reports from the EBMT and the Spanish registry confirmed the efficacy of MYSEC-PM, as well as its superiority over DIPSS or IPSS scores, in predicting posttransplant survival [27, 28]. Of note, about 60% of patients in both these series belonged to the low or intermediate-1 MYSEC-PM risk group, and downgrading from higher IPSS categories was mainly related to the large effect of age in the MYSEC-PM model: in fact, only a minority of patients in a transplant age (i.e., <70 years) are categorized in intermediate-2 or high MYSEC-PM risk. This large contribution of age to the scoring system may thus decrease its sensitivity to the other disease-related adverse risk factors (e.g., circulating blasts), which could be important for transplant decisions [28].

Risk factors for survival after transplant comprise high transfusion requirement, massive splenomegaly, non-sibling donor type [29], advanced age, JAK2 V617F-mutated status, constitutional symptoms [10], and HLA-mismatched donor [11]. Of note, patients in these studies were quite heterogeneous and no unifying prognostic variables can be identified, except for age (>55–57 years). Recently, a new Myelofibrosis Transplant Scoring System (MTSS) was proposed to predict survival after HSCT on the basis of the following clinical and molecular variables: leukocytes >25 × 10^9^/L, platelets <150 × 10^9^/L, Karnofsky scale <90%, age >57 years, ASXL1 mutation (1 point each), JAK2-mutated or triple negative status (2 points), and mismatched unrelated donor (2 points) [27]. According to these variables, patients were stratified in low (score 0–2), intermediate (score 3–4), high (score 5), and very high (score >5) risk groups with a 5-year survival estimation of 83%, 64%, 37%, and 22%, respectively.

**Table:** 

*Prognostic scores: key points*
• New scoring systems have been recently developed for PMF (MIPSS70) and SMF (MYSEC-PM) prognostication, and for transplant outcome (MTTS).
• Still, IPSS and DIPSS are widely used in clinical practice and for the transplant-decision process, although the MYSEC-PM score has been proved to be more accurate in SMF patients.
• Impact of subclonal mutations in SMF deserves confirmation.
• The weight of prognostic variables in patients treated with JAK inhibitors has not been still assessed prospectively.

Besides these prognostic scores, a further element of uncertainty is how to consider the response to ruxolitinib or other JAK inhibitors. It is possible that patients who do not respond to ruxolitinib, even after dose optimization, should be referred early for HSCT. In fact, non-responsiveness to ruxolitinib could eventually be considered as a major selection criterion for referral to HSCT, although more studies are needed to determine the timing at which HSCT should be considered in a non-responsive patient. In a small cohort of patients (*n* = 22) briefly exposed to ruxolitinib before planned HSCT (median treatment duration 97 days, range 20–316), 1-year OS was superior for patients with spleen response to ruxolitinib than for patients who failed or lost their response [30]. In addition, a larger study on 100 patients treated with JAK inhibitors (ruxolitinib 90%, others 10%) showed that 2-year OS ranged from 91% for patients experiencing clinical improvement to 32% for those developing leukemic transformation on treatment with JAK inhibitors; patients with stable disease or transient response had an intermediate prognosis [21]. If a favorable response to JAK inhibitors leads to a better transplant outcome because patients who respond to JAK inhibitors have an intrinsic more favorable biology or because JAK inhibitors ameliorate the clinical status of patients remains an unanswered question.

Finally, we recently reported on a large multicenter cohort of patients treated with ruxolitinib in real life (40% aged <65 years), showing that 18 and 22% of cases were treated for >12 months even with unstable or no spleen response, respectively [31], reflecting a common tendency to use ruxolitinib in clinical practice as a strategy to delay HSCT rather than a bridge to it.

**Table:** 

*Pretransplant ruxolitinib: key points*
• Response to ruxolitinib is associated with favorable outcomes after HSCT.
• Patients failing ruxolitinib therapy should be considered as candidates for HSCT within 6–12 months, if suitable.

### Transplant and posttransplant evaluation: realization, management and outcome

According to the revised 2018 ELN recommendations [32], patients with intermediate-1 risk disease should be considered as eligible for HSCT in the presence of detrimental prognostic factors, such as adverse cytogenetics or mutations [33–36], high need for transfusions [37], or >2% of circulating blasts [38]. All these conditions were met in the present case, except for the lack of information about molecular status when we decided to candidate the patient to HSCT. Prior to HSCT we documented grade 3 fibrosis and osteosclerosis at bone marrow reevaluation, and mutational analysis of the CALR gene revealed a type 2 mutation. This is of interest as patients with mutations in the CALR gene appear to have better overall survival [4] and better posttransplant outcomes [39, 40], even though the prognostic significance of type 2/type 2-like CALR mutations seems to be less favorable than type 1/type 1-like mutations [35, 41]. Furthermore, during the pretransplantation waiting period, the patient’s DIPSS score had raised to 5, and she was thus considered as high risk.

Iron overload is common in MF due to the high requirement for transfusions and the disease-associated inflammatory state; moreover, MF patients have been shown to have higher levels of hepcidin than normal controls, and increased levels of hepcidin and ferritin appear to be a DIPSS-plus-independent adverse prognostic factor for survival [42]. Iron chelation with deferasirox was associated to erythroid improvement and reduction or abolition of transfusion dependence in 15–40% of MF patients [43, 44]. Due to the adverse effects of iron overload on HSCT outcomes [45], evaluation of pretransplant iron status is recommended and treatment with deferasirox may be beneficial in terms of posttransplant engraftment and long-term outcomes [46, 47].

Splenomegaly negatively affects HSCT outcomes as it may be associated with poor graft function and increased mortality [48]. Despite these observations, it remains uncertain if splenectomy prior to transplantation is associated with improved outcomes. At present, there is some evidence to support pretransplant splenectomy [49–51], but postoperative complications, present in around one-half of all patients [52], could lead to delays in HSCT. Therefore, it is accepted that clinical decisions on surgical reduction of spleen size should be made on an individualized basis [19]. In the present case, pre-HSCT splenectomy was not taken into consideration given the presence of clinically significant portal and splenic vein thrombosis, necessitating administration of heparin, and transfusion-dependent anemia.

There is general agreement that, as for other diseases, an HLA-matched donor, either sibling or unrelated, is associated with superior overall survival [27, 53], although some experiences found a worse outcome in MF patients transplanted from an unrelated donor, regardless of HLA-matching status [54]. However, there is increasing evidence about the use of alternative donors. Raj et al. reported on 56 MF patients transplanted between 2009 and 2015 from a family mismatched donor (i.e., relatives with ≥2 Ag HLA different from recipient), with a 2-year overall survival rate of 56% but a rather high non-relapse mortality (NRM) of 38% at 2 years [55]. In a recent retrospective review, NRM and 4-year OS rates were similar in patients receiving their first transplant from HLA-matched related donor peripheral blood stem cells or from HLA-MUD bone marrow, while long-term survival was inferior in patients transplanted from umbilical cord blood, partly due to higher NRM [56]. Information about haploidentical related donor in MF is still scarce; in a comparison between HLA-identical sibling and “alternative” donors, including 20 haploidentical, transplanted in the years 2011–2014, Bregante et al. found similar survival rates in the two cohorts (72% and 69%, respectively) [57].

With regards to the conditioning regimen, there are limited data on the choice of the optimal regimen give the lack of prospective clinical trials comparing MAC to RIC in MF [58]. However, retrospective studies in the pre-ruxolitinib era have reported that ideal candidates for MAC are younger patients (<40 years old), without comorbidities and with HLA-identical sibling donor [59], while RIC may be preferred in patients older than 50 years [4, 58, 60]. Accordingly, RIC with fludarabine + thiotepa was adopted in the present case. A recent prospective randomized study compared fludarabine in combination with busulfan or thiotepa as conditioning regimen in 60 patients undergoing HSCT for MF, showing comparable clinical outcome in the two arms [51]. However, other available data are somewhat discrepant on the outcomes of patients undergoing fludarabine-melphalan based RIC regimens [54, 61]. Recently, Gupta et al. reported a prospective trial of ruxolitinib treatment followed by a RIC regimen: the cumulative incidences of graft failure, NRM, acute GvHD, and chronic GvHD at 24 months were 16%, 28%, 64%, and 76%, respectively; 2-year overall survival was 61 and 70% for patients receiving transplant from related or unrelated donor, respectively. The role of pretransplant ruxolitinib is also supported by a retrospective study of 159 MF patients receiving (29%) or not receiving ruxolitinib (71%) at any time before HSCT: graft failure, time to engraftment, and NRM were similar, while a trend for lower risk of relapse was seen in the ruxolitinib group [62]. Moreover, there was no difference in any outcome variable between those who responded to ruxolitinib and those who failed or lost response to the drug [62]. Another aspect that remains unclear is the rate and schedule for pretransplantation discontinuation of ruxolitinib. In our case, ruxolitinib was quickly tapered downward at 1 week prior to HCT and stopped the day before starting the conditioning regimen. This decision was largely made upon empirical considerations and on the observation that following interruption or discontinuation of ruxolitinib symptoms of MF may return over a period of ~1week [63]. Some studies have suggested that ruxolitinib should be continued near to the start of conditioning therapy [30, 53, 60], while a recent experience reported the safety of continuing low-dose ruxolitinib (5 mg BID) until stable engraftment in 12 MF patients undergoing allogeneic transplant [64]. Drug discontinuation symptoms were reported to be more common in patients who had a longer interval between last dose of a JAK1/2 inhibitor and beginning of the conditioning regimen, with 29% of patients developing symptoms with an interval ≥6 days compared with only 7% among those with an interval <6 days [53]. Moreover, the possible appearance of significant clinical events related to discontinuation of ruxolitinib may occur, which may even lead to delay in HSCT [53].

**Table:** 

*Transplant procedure: key points*
• HLA-matched sibling or unrelated donor is the preferable choice, but there is increasing evidence of feasibility of HSCT form alternative donors.
• Patients older than 50 years should receive a RIC transplant.
• The conditioning regimen is based on fludarabine combined with either busulfan, thiotepa or melphalan.
Patients receiving pretransplant ruxolitinib should be tapered and discontinued just before starting the conditioning regimen to avoid a cytokine rebound.

Lastly, the role of ruxolitinib post HSCT remains unclear and there is very limited information to guide clinicians. The presence of minimal residual disease (MRD) or overt relapse after HSCT pose a significant clinical challenge, and MF recurrence remains the main cause of death in the long term, as has been recently demonstrated in a large retrospective analysis on over 1000 patients with MF transplanted between 1995 and 2014 in Europe [65]. Detection of posttransplant relapse may be challenging, as bone marrow fibrosis may persist for months after HSCT [66] and molecular monitoring is not yet standardized. While detection of JAK2 mutations after transplant has been found to predict MF relapse [67], no data are available on the role of CALR, MPL or monitoring other molecular markers and their role in guiding therapy. In view of the lack of uniform criteria, we propose serial monitoring (e.g., at 3, 6, and 12 months after HSCT) of bone marrow biopsy and driver mutations present in individual MF patients.

In patients relapsing after transplant, discontinuation of immunosuppressive drugs, administration of donor lymphocyte infusion (DLI), chemotherapy, and second allogeneic transplant has been proposed [68]. Response to DLI seems to be more effective in patients with recurrence of MRD and monitored by JAK2 mutation levels in peripheral blood than in patients with full clinical relapse [69]. A role of JAK inhibitors in this setting appears reasonable, but still scarcely defined [70]. More recently, ruxolitinib has also emerged as an option for treating steroid-resistant acute and chronic GVHD [71], and promising results have been reported in a small group of MF patients who were treated with low-dose ruxolitinib until stable engraftment [64].

**Table:** 

*Posttransplant management: key points*
• Albeit the limited experience, there seems to be a role for ruxolitinib in the peri- and posttransplant period.
• Optimal histological and molecular monitoring of MF after HSCT is still to be defined.

## Conclusion

HSCT remains the main curative option for MF patients even in the era of JAK inhibitors. Eligibility for transplant should rely on the integrated evaluation of disease- and transplant-related risk factors, particularly in older adults. A thorough mutational analysis covering driver and subclonal mutations may help to define indication for HSCT in lower-risk fit patients. RIC nowadays represents the most common type of HSCT in MF, and busulfan or thiotepa plus fludarabine are both appropriate conditioning regimens. Response to ruxolitinib as a “bridge to transplant” can be obtained in about 30–50% of patients and predicts a favorable outcome after HSCT.

Some of the most pressing issues to be studied in future prospective clinical trials involve the place and duration of JAK inhibitors in the pretransplant period (together with an optimized timing sequence for discontinuation), early versus late transplantation, and the role of ruxolitinib or other drugs in managing patients in the posttransplant period.
